# Outcome of therapy-related myeloid neoplasms treated with azacitidine

**DOI:** 10.1186/1756-8722-5-44

**Published:** 2012-08-01

**Authors:** Luana Fianchi, Marianna Criscuolo, Monia Lunghi, Gianluca Gaidano, Massimo Breccia, Alessandro Levis, Carlo Finelli, Valeria Santini, Pellegrino Musto, Esther N Oliva, Pietro Leoni, Antonietta Aloe Spiriti, Francesco D’Alò, Stefan Hohaus, Livio Pagano, Giuseppe Leone, Maria Teresa Voso

**Affiliations:** 1 Istituto di Ematologia, Università Cattolica del Sacro Cuore, 00168, Roma, Italy; 2 Ematologia, Dipartimento di Medicina Traslazionale, Università del Piemonte Orientale Amedeo Avogadro, Novara, Italy; 3 Ematologia, Università La Sapienza, Roma, Italy; 4 Ospedale di Alessandria, Alessandria, Italy; 5 Universita' di Bologna, Bologna, Italy; 6 Ematologia Firenze, Firenze, Italy; 7 Dipartimento Onco-Ematologico, IRCCS, Centro di Riferimento Oncologico della Basilicata, Rionero in Vulture, Italy; 8 Azienda Ospedaliera Bianchi-Melacrino-Morelli, Reggio Calabria, Italy; 9 Ematologia Ancona, Ancona, Italy; 10 Ospedale Sant’Andrea, Roma, Italy

**Keywords:** Therapy related myeloid neoplasms, Hypomethylating agents

## Abstract

**Background:**

Therapy-related myeloid neoplasms (t-MN), including myelodysplastic syndromes and acute myeloid leukemia (t-MDS and t-AML) are associated to clinical and biologic unfavorable prognostic features, including high levels of DNA methylation.

**Methods:**

We retrospectively evaluated 50 t-MN patients (34 MDS and 16 AML) selected among all patients receiving azacitidine (AZA) at 10 Italian Hematology Centers. Patients had developed a t-MN at a median of 6.5 years (range 1.7- 29) after treatment of the primary tumor (hematological neoplasm, 27 patients; solid tumor, 23 patients).

**Results:**

The overall response rate was 42% (complete remission: 10 patients, partial remission: 2 and hematological improvement: 8 patients) and was obtained after a median of 3 cycles (range 1–6). Median overall survival (OS) was 21 months (range 1–53.6+) from AZA start. OS was significantly better in patients with less than 20% blasts, in normal karyotype t-AML and when AZA was used as front-line treatment. This was confirmed by the multivariate analysis.

**Conclusions:**

This study reports efficacy of AZA in the largest series of therapy-related MN patients treated with 5-AZA. Our data show that blasts and karyotype maintain their important prognostic role in t-MN also in the azacitidine era.

## Background

Therapy-related myeloid neoplasms (t-MN) have been recognized as a clinical and biological distinct entity in the 2008 WHO AML classification [1]. By definition, these diseases occur after cytotoxic treatment for a primary cancer or after immunosuppressive treatment. t-MN account for about 10% of all AML, and may arise from few months to several years after the primary tumor, depending on type of cytotoxic treatment, cumulative dose and dose-intensity [2,3]. At disease onset, t-MN typically present with unfavorable features, such as peripheral blood cytopenias, complex karyotype, and chromosome 11, 5 or 7 abnormalities [4-6]. Increased rates of gene-specific hypermethylation have been reported in t-MN [6-9].

Because performance status is often poor, mostly due to age, primary tumor and exposition to prior therapies, the use of standard chemotherapy protocols is generally impaired in these patients. Treatment varies from supportive care to conventional chemotherapy and allogeneic HSCT, but outcome remains dismal. Even in aggressively treated cases, complete remission rates are usually lower and remission duration is shorter than in *de novo* MDS/AML, with median survival rates of less than one year in most studies [5,10-13]. Allogeneic HSCT is the only curative option, but it is not feasible in the majority of patients and is often complicated by high transplantation-related mortality rates [10]. Response rates similar to *de novo* AML have been recently obtained only for t-AML patients carrying t(15;17) or t(8;21) translocations and included in standard protocols [5].

During the last few years, hypomethylating agents have been largely used in the treatment of intermediate-2 and high risk MDS [14]. Azacitidine has been shown to be effective also in unfavorable patient sub-groups, inducing response rates up to 60% and improving survival compared to conventional care [14-16].

Here we report that azacitidine treatment might represent a safe and effective option in t-MN patients, given the biological and clinical characteristic of the disease and the potentially fatal consequences of more aggressive therapies.

## Methods

### Patients and methods

In this multicenter study, we retrospectively collected clinical data of patients diagnosed with t-MN and treated with azacitidine (AZA, Vidaza^TM^, Celgene Corp.) at 10 Italian Hematology Centers. Fifty cases of t-MN consecutively treated with AZA were identified and analyzed among all patients with MDS or AML receiving AZA at the participating centers between October 2005 and August 2011. Criteria for AZA treatment were: diagnosis of t-MN according to the WHO classification, defined as leukemias occurring in patients with a history of prior cytotoxic treatment for a primary tumor. Further criteria were adequate renal and hepatic function, and absence of uncontrolled infections. Patients gave written informed consent to treatment and to the collection of clinical data, in accordance with the Declaration of Helsinki and institutional guidelines.

AZA was started at a median of 1.8 months (range 0–29) from t-MN diagnosis, at the conventional dose of 75 mg/m^2^ daily for 7 days (36 patients, 72%), or at a fixed dose of 100 mg daily for 5, 7 or 10 days (3, 7 and 4 patients, respectively) every 4 weeks. A median of 4 cycles (range 1–23) were administered, with 37.7% of patients receiving 4 or more cycles.

AZA was administered until disease progression, unacceptable toxicity, or patient decision to withdraw consent. Response was assessed according to the modified International Working Group (IWG-2006) criteria [17,18]. We evaluated overall response (OR), including complete remission (CR), partial remission (PR) and hematological improvement (HI) rates, and overall survival.

Adverse events were graded according to the National Cancer Institute Common Toxicity Criteria (CTC-NCI, version 4.0).

### Statistical analysis

Associations between patient characteristics were analyzed using the Fisher’s exact test. Overall survival was calculated from start of AZA treatment to date of death from any cause or of the last follow-up. Survival curves were estimated using the Kaplan-Meier product limits method. Log-rank test was applied to study survival differences according to patient characteristics. Age (≤ 65 vs >65 y.o.), primary malignancy (hematological vs solid), comorbidity index (HCT-CI) (19), AZA dose (75 mg/m^2^/7 days vs 100 mg/day 10 days vs 100 mg/day 5 or 7 days), WHO diagnosis (RCMD vs RAEB 1/2 vs AML), karyotype (normal vs abnormal), chromosome 7 abnormalities, transfusion dependence, previous treatment (no vs ESA vs hydroxyurea or chemotherapy) were evaluated in the univariate analysis. Cox proportional hazard model was also used for multivariate analysis of factors with prognostic significance in the univariate analysis (blast count >20%; karyotype, previous cytotoxic treatment for t-MDS/AML). Computations were performed using the Stata 10.0 software (Stata Corp., College Station, TX). All tests were two-sided with α = 0.05.

## Results

### Patient characteristics

The main patient characteristics at diagnosis are reported in Table 1. There were 28 males and 22 females, with a median age of 66 years (range 37–84). According to the WHO classification, there were 34 MDS (12 RCMD, 9 RAEB1 and 13 RAEB2) and 16 AML.

**Table 1:** Clinical characteristics of 50 patients with t-MN treated with azacitidine

**Patient Characteristics**	***n (*** **range)**
**Median age** (years, median, range)	66 (37–84)
**Sex** (M/F)	28/22
**ECOG PS**	
0–1	46
2-3	4
**Type of t-MDS/AML according to the WHO**	
- AML:	16
BM-blasts 20-29%	5
BM-blasts ≥30%	11
- MDS:	34
RCMD	12
RAEB1	9
RAEB 2	13
**Primary malignancy :**	
- Lymphoproliferative disease	19
- Multiple myeloma	3
- Chronic myeloproliferative disease	5
- Breast cancer	6
- Urogenital	9
- Other	8
**Treatment for Primary Malignancy:**	
- Chemotherapy	27
- Radiotherapy	9
- RTx + CTx	14
**Median latency** between primary cytotoxic therapy and t-MN diagnosis (years)	6.1 (0.2-29.8)
**Median value at diagnosis (range):**	
- White blood cell counts (10^9^ /L)	2.6 (0.1-26)
- Platelets (10^9^ /L)	69.5 (5–395)
- Bone Marrow blasts (%)	13 (1–90)
- LDH (UI/L)	403 (130–2498)
**Median interval** between t-MN diagnosis and AZA treatment (months)	1.8 (0–39)
**Response to treatment:**	
**- Overall response:**	20 (42%)
- CR	10 (21%)
- PR	2 (4.2%)
- HI	8 (16.7%)
- Stable disease	15 (31%)
- Progression	13 (27%)
- Not evaluable	2
**Median cycles** number for response	3 (range 1–6)

Legend: *RTx*: Radiotherapy ; *CTx*: Chemotherapy; *CR*: complete remission; *PR*: partial remission; *HI*: hematological improvement.

All patients had previously received chemotherapy (27 patients, 52%), radiotherapy (9 patients, 17%) or a combination of both (14 patients, 27%) for their primary malignancy.

The primary malignancy was a hematological neoplasm in 27 cases (54%), and a solid tumor in 23 cases (46%). t-MN occurred at a median of 6.1 years (range 0.2 - 29.8 years) from treatment of the primary malignancy (Table 1).

Karyotype was evaluable in 47 patients and was normal in 17 (34%), complex (3 or more aberrations) in 20 (43%), and with a single chromosome abnormality in 10 cases (20%). Deletion or monosomy of chromosome 7 was present as single alteration or in the context of a complex karyotype in 14 patients (30%).

Twenty-six patients (52%) were transfusion-dependent before AZA start [median 2 units of red blood cells (range 1–10) /month and median 5 units of platelets /month (range 1–12)]. Four patients had previously received erythropoietin, and 6 patients had previously been treated with hydroxyurea or standard AML induction therapy, while in 40 patients AZA was used as front-line treatment.

### Treatment response and overall survival

Treatment response was evaluated in 48 patients (35 MDS and 13 AML) after a median of 3 cycles of AZA (range 1–6). Two patients received only 1 cycle and interrupted treatment on patient’s request (1 patients with stable disease) and due to severe constipation (1 patient). (Table 1). The overall response rate (ORR) was 42%, with complete remission in 10 patients (21%), partial remission in 2 (4.2%) and hematological improvement in 8 patients (16.7%). The disease was stable in 15 patients (31%), while 13 patients (27%) presented disease progression at a median of 4 AZA cycles (range 3–7). Treatment was generally well tolerated. Sixteen patients (32%) experienced grade 3 or 4 myelosuppression including neutropenia (8 patients), thrombocytopenia and neutropenia (7 patients) or anemia (1 patient). Eight patients (16%) developed an infection (pneumonia in 7 patients and sepsis in 1), that was the cause of death in 2 cases (4%). Other grade 3 or 4 non hematological adverse events consisted of erythema at the injection site in 2 cases and constipation in 2 cases.

Median duration of response was 7 months (range 3-47+). Five patients (10%), 4 resistant to AZA, underwent allogeneic stem cell transplantation following azacitidine.

Notably, 5 patients (10%) presented a relapse of the primary malignancy (1 non-Hodgkin lymphoma, 2 breast cancers, 1 bladder cancer and 1 cancer of the uterus) after a median of 4 cycles of AZA (range 2–10), and at a median of 13 years from the diagnosis of the primary malignancy (range 9–26 years). None of the patients died as a consequence of these relapses, but they were resistant to AZA and died due to t-MN progression.

**Figure 1:**
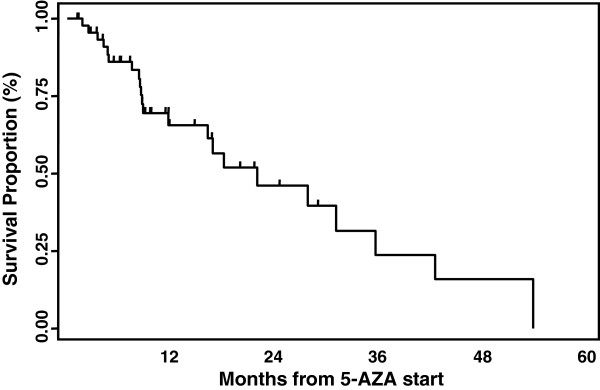
**Overall survival of 50 t-MN patients treated with azacitidine**. Median survival was 25.6 months (range 1.1-61.1+) from initial diagnosis and 21 months (range 1–53.6+) from AZA start

Median overall survival of t-MN patients was 25.6 months (range 1.1-61.1+) from diagnosis and 21 months (range 1–53.6+) from azacitidine start (Figure 1). Survival was 72% (95 C.I.: 53-82%) at the median follow up of 8.7 months (range 1–54).

**Figure 2:**
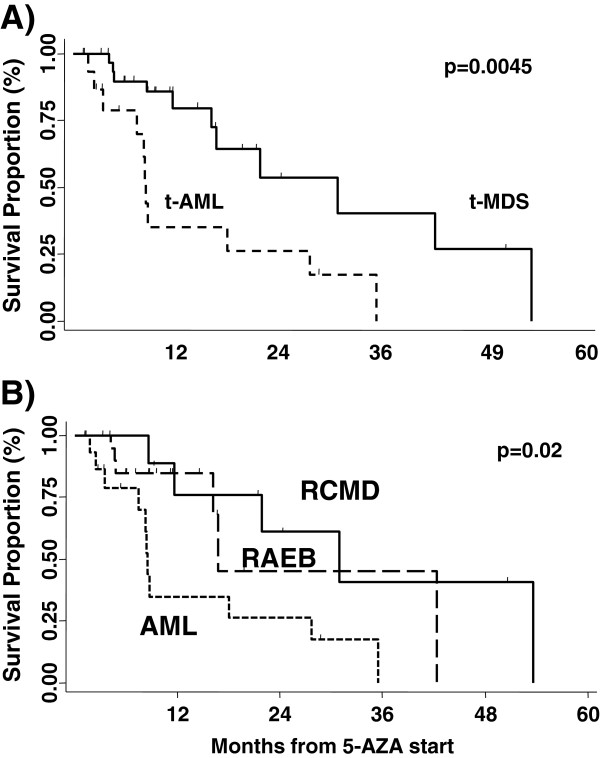
**t-AML patients had a significantly worse survival than t-MDS patients.A**) Median survival was 8.5 months in t-AML versus 30.9 months in t-MDS (p = 0.0045), classified according to the WHO classification (over 20% bone marrow blasts as definition for AML) **B**) In t-MDS, there were no survival differences when stratifying for WHO subtypes (RCMD vs RAEB1-2)

**Figure 3:**
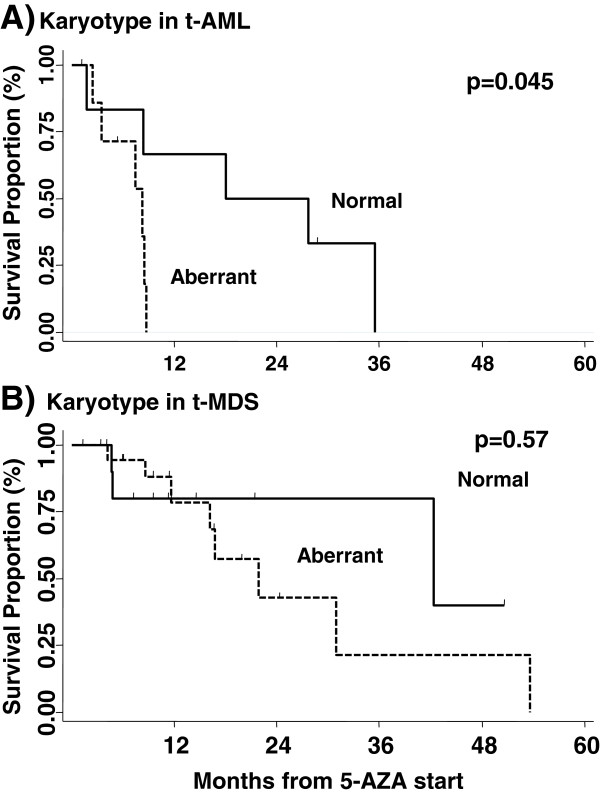
**Aberrant karyotype was predictor of death in t-AML, but not in t-MDS patients.** Survival was studied according to the presence of any cytogenetic aberrations versus normal karyotype

By stratifying patients according to t-MN type (below or over 20% bone marrow blasts), the overall response rate appeared to be higher in t-MDS versus t-AML, albeit the difference was not statistically significant (50% vs 21%, p = 0.1). Overall survival from initial t-MN diagnosis or from AZA start was significantly better in t-MDS versus t-AML (40.4 months versus 19.4 months from initial diagnosis, p: 0.005, and 30.9 months versus 8.5 months, from AZA start, p: 0.0045, Figure 2). This significant difference in overall survival was confirmed also when stratifying patients according to WHO categories (RCMD versus RAEB1-2 versus AML, p = 0.02) (Figure 3).

Concerning significant prognostic factors for survival, aberrant karyotype confirmed its negative value in t-AML (median survival: 8.3 vs 17 months in normal karyotype, p = 0.045), but not in t-MDS (median survival: 21.9 vs 42.3 months, p = 0.57, Figure 3). Survival was superior in patients who received AZA as front-line treatment, compared to patients who received other cytotoxic drugs to treat t-MN prior to AZA (p = 0.0001, Table 2). No survival differences were observed when stratifying patients according to the type of previous malignancy, time between treatment of the primary malignancy and development of t-MN, time between t-MN diagnosis and AZA start, comorbidity index (HCT-CI) [19] peripheral blood counts, IPSS score in MDS, transfusion-dependence or AZA dose (Table 2). The multivariate analysis confirmed the significant negative prognostic value of bone marrow blast over 20%, and karyotype (Table 3).

**Table 2:** Prognostic factors for overall survival

	***Patients*** ***(n)***	***Number of responders***^***§***^	***Median OS*** ***(months)***	***p***
**Age**				
≤ 65 years	24	10	21.9	0.85
> 65 years	26	10	24.5	
**Primary Malignancy**				
Hematological	27	12	11.67	0.83
Solid	23	8	17.97	
**azacitidine dose**				
75 mg/smq/7 days	36	12	16.7	0.31
100 mg/day 10 days	4	3	14.2	
100 mg/day 5 or 7 days	10	5	16.2	
**WHO diagnosis**				
RCMD	12	9	30.9	
RAEB 1/2	22	7	16.8	**0.02**
AML	16	4	8.5	
**Karyotype**				
Normal	8	8	27.7	
Single or double abnormality	9	3	11.6	0.66*
Complex	20	9	16.2	
**Chromosome 7 abnormalities**				
Yes	14	8	11.63	0.72
No	33	12	17.96	
**Transfusion dependence**				
Yes	26	10	11.6	0.38
No	24	10	21.9	
**Previous Treatment**				
No pre-treatment	40	17	27.7	**0.0001**
ESA	4	2	16.2	
Hydroxyurea	2	1	9.5	
Conventional Chemotherapy	4	0	5.4	
**HCT-CI**^**#**^				
0–2	5	0	8.8	
3	19	6	8.5	0.2
4	6	4	53.7	
>5	9	4	16.2	

* The difference in overall survival between normal and aberrant karyotype was however statistically significant restricting the analysis to t-AML patients (Figure 3A).

^***§***^ Number of patients achieving CR, PR or HI; ^#^ HCT-CI data were available for 39 patients.

**Table 3:** Multivariate analysis for overall survival

	**Hazard Ratio**	**p**	**95% C.I.**
**WHO type (t-AML vs t-MDS)**	3.49	0.03	1.17-10.46
**Karyotype (aberrant versus normal)**	2.2	0.015	0.75-6.58
**Front-line AZA (no vs yes)**	3.45	0.08	0.87-13.62

## Discussion

Prognosis of patients with therapy-related myeloid neoplasms treated with conventional chemotherapy is poor, with lower remission rates and shorter remission duration than in *de novo* AML. Furthermore, t-MN display high levels of DNA methylation [6-9] and high frequency of monosomal karyotype, including complete or partial deletions of chromosome 5 or 7 [5,10,20].

The biological features and the unsatisfactory results of standard chemotherapy provided the rationale for our retrospective study on the role of azacitidine in t-MN. We observed 42% overall response rate and a median overall survival of 21 months. The outcome of our AZA-treated t-MN patients is similar to results obtained in *de novo* high-risk MDS treated with AZA at standard doses [14,21] and also favorably compares to conventional therapy in t-MN [10,12]. In a recently published study by Kayser et al.. on 200 selected t-AML patients included in 6 prospective German-Austrian multi-center trials, complete response to standard induction therapy was 63% in t-AML, with a median overall survival of about 15 months [5]. There are only few data on AZA efficacy in t-MN. Seventy-four t-MDS were included in a large cohort of 282 high-risk MDS treated with AZA [15]. ORR was 43% for the whole patient cohort, but therapy-related forms had a shorter survival (median 9.2 months versus 15.3 months in *de novo* MDS, p = 0.002) [15].

In our study, AZA efficacy was particularly evident in t-MDS, who had a significantly better overall survival than t-AML patients. This was true also considering WHO subgroups, with significantly different overall survival in refractory cytopenia with multilineage dysplasia (RCMD), versus RAEB-1 and −2, versus AML. These data, confirm the role of WHO subgroups in *therapy-related* as in *de novo*-MDS [22,23].

Although karyotype maintains its prognostic value in t-AML, survival of t-AML patients within the same cytogenetic risk group is generally shorter than that of patients with *de novo* AML [24]. Karyotype prognostic groups differ between MDS and AML. Due to the limited number of patients, especially those with a favorable karyotype, we grouped patients according to the type of t-MN and presence of any chromosomal abnormalities. As a result, our t-AML patients with aberrant karyotype (including chromosome 7 alterations in 30% of cases) had a significantly shorter survival than patients with a normal karyotype. Also, patients treated with AZA front-line had a significantly better outcome than patients who received previous cytotoxic drugs to treat t-MN. In the same line, normal karyotype, bone marrow blast counts below 15% and no previous treatment with low-dose cytosine-arabinoside independently predicted better response to AZA in higher risk MDS [15]. It is also possible that patients treated with cytoreductive chemotherapy had a more aggressive t-MN disease, and thus were less likely to respond to azacitidine when used in second line.

In our series 5 patients had a chronic myeloproliferative disease as previous neoplasm. The inclusion of these disorders into the list of primary malignancies is debated due to possibility that the AML transformation might represent a natural evolution of disease. The exposure to cytotoxic agents however could hasten or induce this transformation as also suggested by an increased risk of AML in patients who were treated with radioactive phosphorus or chlorambucil in the Polycytemia Vera Study Group trial [25].

Five of fifty patients had a relapse of the previous malignancy during treatment with AZA after a median of 4 cycles, and at a median of 13 years from the diagnosis of the primary malignancy. This observation should be at least a warning to include re-evaluation and monitoring of disease activity of the primary malignancy in future studies on t-MN treatment. There are only very limited data in the literature. More than 20 years ago, Carr et al. reported a higher incidence of testicular tumors after prolonged treatment with AZA in a rat model with an already high spontaneous tumor rate [26]. Itzykson et al did not report any relapse of the primary tumor in 74 t-MDS patients treated with a median of six AZA cycles [15]. On the other hand, AZA efficacy has been reported in solid tumors and lymphoproliferative diseases [27,28]. Our group has recently reported a patient with Hodgkin lymphoma and a t-MDS, who achieved complete response of MDS and HL following AZA treatment [29].

In conclusion, our data indicate effectiveness of up-front AZA in t-MN, and are particularly encouraging in the setting of t-MDS. Blasts and karyotype maintain their important prognostic role for t-MN also in the azacitidine era. As there is no accepted standard treatment for patients with t-MN, other treatment approaches in the same time period of our study varied from supportive care only to low-dose cytarabine, gemtuzumab-ozogamicin in combination with G-CSF and cytarabine, standard induction chemotherapy and front-line allogeneic bone marrow transplantation [30]. Only about 25% of t-MN patients registered in 4 of the participating centers were treated with azacitidine. These data will help to design prospective studies for t-MN to address efficacy and risks of AZA treatment, in particular as a less toxic “bridging” therapy to a curative treatment approach as allogeneic transplantation.

## Competing interests

The following authors declared a conflict of interest with Celgene: Honoraria: C.F., A.A.S., G.L., M.T.V.; Consultancy: A.L., E.O., P.M.

## Authors’ contribution

LF collected and analyzed patients’ data, and wrote the manuscript. MC collected and analyzed patients’ data. ML collected and analyzed patients’ data. GG collected and analyzed patients’ data, and revised the manuscript. MB collected and analyzed patients’ data, and revised the manuscript. AL collected and analyzed patients’ data, and revised the manuscript. CF collected and analyzed patients’ data, and revised the manuscript. VS collected and analyzed patients’ data, and revised the manuscript. PM collected and analyzed patients’ data, and revised the manuscript. ENO collected and analyzed patients’ data. PL collected and analyzed patients’ data. AAS collected and analyzed patients’ data. FD collected and analyzed patients’ data. SH collected and analyzed patients’ data, and revised the manuscript. LP collected and analyzed patients’ data. GL collected and analyzed patients’ data, and revised the manuscript. MTV analyzed patients’ data, and wrote the manuscript. All authors read and approved the final manuscript.
